# Over-Feeding and Summer Complaint

**Published:** 1884-06

**Authors:** David Little

**Affiliations:** Rochester, New York


### OVER-FEEDING AND SUMMER COMPLAINT.

DAVID LITTLE, M. D., ROCHESTER, NEW YORK,

*Visiting Physician to the Rochester Orphan Asylum.*

The object of the little I have to say is not to propound any new thing pertaining to the nature, cause, prevention, or cure of the enteric diseases included under the popular name, summer complaint.

It is solely to emphasize a single factor in their production, under the belief that by giving it the prominence it deserves a rational way will readily suggest itself of meeting and repulsing this slaughterer of the innocents.

It is unnecessary for my present purpose to describe the various digestive disorders that are comprehended under the name of summer complaint.

Attention is simply called to the conditions that obtain in their causation. Text-books and the common experience of physicians give for answer:

Urban life, artificial food, summer heat.

Of course, each one of these three includes and entails a multitude of debilitating agencies, but in the main they cover the whole ground.

They make frightful havoc, and when we consider how, acting together, these allied forces of evil are multiplied and intensified in their maleficence, we can only wonder that so many escape their onslaught.

Moralists tell us the first step downward is the one to guard against, and this is true physically as well as morally.

This brings us to the pith, how to guard against indigestion. Answer, *avoid over-feeding.* To avoid wrong or improper feeding goes without the saying; medical books and medical heads are full of it, and as a result, infant foods of numberless designs and kinds are concocted, advertised, and given, with various results.

Each physician in each case must, taking human milk as his model, get as near to nature as possible, and "fight it out on this line if it takes all summer" (and it generally does).

Over-feeding, it seems to me, in military parlance, is the key to the position. This, in my belief, is the bane of bottle-fed children.

Look at it: The doctor is called to a case of summer diarrhœa. He prescribes, and leaves instructions as to what food to give and how often, and adds, "Keep the child well aired, clean, cool, and quiet," and goes on his way, thinking he has been specific enough.

Now, what does the child's attendant do? That last injunction about keeping the child quiet makes a major impression, because this same quiet consorts with her own comfort. The child cries and must be quieted, and the ready bottle is its comforter. Through the day that other injunction about feeding only so often acts in a measure as a deterrent; but the long night comes, and the tired nurse or mother needs quiet, too, and now the bottle becomes a duplex comforter. Filled and refilled, it is kept to the child's lips.

A stomach that has no rest gives up, or gives out.

A fundamental principle in the treatment of disorder of any organ is to give it a rest. In the case of digestive misdemeanors nothing is so effective as to starve the offending viscera into good behavior. And this plan, at first thought so abhorrent to the fond mother, or indulgent (*or indolent*) nurse, may be made feasible by a simple explanation. Tell her the child cries more from thirst than from hunger. Lay down inflexible rules about amount of food and times for feeding, but give her *carte blanche* to water the infant as often as it cries. Tell her hot weather induces perspirations, and that as surely and in the same way as waste makes wants, sweating makes thirst.

This sounds plausible, but the doctor of to-day is a skeptic and wants evidence. My experience is limited and my figures are few, but it is believed that they are significant if not convincing.

For twenty-one years I have been a physician to the Rochester Orphan Asylum. Each of these years had witnessed deaths from enteric diseases until 1882.

In the early summer of that year I said to the matron, "Feed your babes but once in three hours during the day, but give them water to drink as often as they will take it."

The summer came and went, and when frosts appeared I congratulated her on the good results of our plan; not a child had died, and no serious case of diarrhœa had occurred.

"Yes," said she, "but it seemed cruel to feed these babes but three times a day."

*She had actually carried out my instructions to the letter as she had misunderstood them.* Instead of once in three hours, she thought I had said three times a day!

On the following (that is, last) summer the plan was carried out, giving the infants food once in three or four hours, according to age, during the day, and an additional meal in the night if the child awoke and would not be quieted with a simple drink of water.

The same immunity from summer complaints ensued as in the previous year, with two exceptions, and these exceptions emphatically proved the rule.

They were children of tubercular parents, and because of scrofulous manifestations were removed to the hospital, then empty, in the upper story of the building. A nurse was detailed from the hired help. Soon these infants became dyspeptic, intractable gastro-enteritis followed, and in a few days they died.

Inquiry elicited the fact that the newly made nurse had kept a bottle every night and all night in their mouths, for, as she declared, she could have no peace without. In legal parlance, it is submitted that a case is made. That is, that rest for the stomach may be obtained by recognizing thirst more than hunger as a summer want, and thus, by prolonging the intervals of feeding, preventing indigestion and its deadly train of attendants.—*Am. Journal of Obstetrics, etc.*

